# Real-time traffic signal optimization for urban mobility: a reinforcement learning-enhanced framework with application to Kuwait City

**DOI:** 10.3389/frobt.2025.1669952

**Published:** 2025-09-24

**Authors:** Abedalmuhdi Almomany, Eedi Eedi, Muhammed Sutcu

**Affiliations:** 1 Department of Electrical and Computer Engineering, Gulf University for Science and Technology, Hawally, Kuwait; 2 Department of Engineering Management, GUST, Hawally, Kuwait

**Keywords:** smart cities, traffic signal control, field programmable gate Array(FPGA), max-pressure Algorithm’ delay-based optimization, intelligent transportation systems (ITS), real-time traffic management

## Abstract

**Introduction:**

This study develops an intelligent, adaptable traffic control strategy using advanced management algorithms to enhance urban mobility in smart cities. The proposed method aims to minimize wait times, reduce congestion, and improve environmental health through better traffic management.

**Methods:**

The approach thoroughly investigates and evaluates rule-based (Fixed-Time), optimization-based (Max-Pressure and Delay-Based), and machine-learning–driven (Reinforcement Learning) algorithms under various traffic conditions. This enables the system to automatically select the algorithm that most effectively minimizes wait times and reduces traffic congestion. Microscopic traffic simulations are employed to test the system, and various statistical analyses are conducted to evaluate performance. A Reinforcement Learning (RL) variant is further utilized to validate the method's effectiveness against alternative approaches.

**Results:**

The selected algorithms are executed on high-performance Field Programmable Gate Array (FPGA) platforms, which are suitable for embedded, energy-constrained smart city environments due to their lower latency and power consumption compared to general-purpose GPUs. The proposed system achieves a speedup of over 7× compared to modern high-speed general-purpose processing units (GPPUs), demonstrating the efficiency of the custom FPGA-based pipelined architecture in real-time traffic management applications.

**Discussion:**

The method not only improves traffic flow but also significantly reduces fuel consumption and carbon dioxide emissions. This study further explores how the proposed solution can be leveraged to address Kuwait’s significant traffic challenges and contribute to improving air quality in the region.

## 1 Introduction

Smart cities are gaining popularity as a way to utilize new technologies to improve the lives of people living in cities by managing city infrastructure in an efficient manner. As cities expand and more people own cars, traffic jams become increasingly common, making life more complicated and exacerbating air pollution, greenhouse gas emissions, and fuel use. Recent technology advancements can be utilized effectively to address the traffic management issue and achieve the goals of preserving the environment, improving the air quality and public health, and boosting economic productivity (Michailidis et al., 2025), (Ault and Sharon, 2021).

Fixed-time plans and other traditional methods of controlling traffic signals do not always function effectively in cities, as traffic is constantly changing, leading to less efficient intersection operations and longer lines of cars (Banerjee, 2024). Researchers have developed advanced traffic signal control strategies that combine ideas from traffic flow theory, mathematical optimization, and, increasingly, artificial intelligence techniques (Michailidis et al., 2025), (Alvarez Lopez et al., 2019). These strategies address these problems by constructing an efficient real-time solution that has the capability to overcome the issues of road queues, including lengthy and undesirable delays. However, researchers and practitioners still struggle to consistently achieve near-optimal control in a wide range of changing and complex scenarios as urban traffic systems become more complicated and larger (Ault and Sharon, 2021), (Ayeelyan et al., 2022).

This study examines a set of state-of-the-art algorithms for traffic signal control, such as Fixed-Time, Max-Pressure, Delay-Based, and the Hybrid Delay approach, under various demand scenarios, utilizing microscopic traffic simulation models (Alvarez Lopez et al., 2019). To further expand the limits of adaptive traffic control, we also examine a type of reinforcement learning (RL). This technique can learn optimal policies by interacting with the traffic environment in real time (Michailidis et al., 2025), (Ault and Sharon, 2021), (Ayeelyan et al., 2022). By comparing these algorithms, we can gain insight into their strengths and weaknesses, which enables us to select the most effective control strategy for a given traffic condition.

While the software-level algorithm introduces improvements at the traffic-responsive level, high-demand urban corridors introduce more timing challenges. To achieve the desired level of real-time requirement, high-speed hardware computation devices can be used, such as the field programmable gate arrays (FPGAs) platform (Almomany and Jarrah, 2024), (Jarrah et al., 2022a), (Jarrah et al., 2022b). Delays in making decisions, even as short as a few milliseconds, can lead to significant difficulties at busy intersections (Banerjee, 2024), (Helbing et al., 2000). This study also investigates the possibility of implementing these algorithms on the FPGA devices, which can process data in parallel to get decision cycles with very low latency (Banerjee, 2024). FPGA technology not only speeds up computations but also makes it possible to deploy intelligent transportation systems in a way that is scalable and uses less energy (Almomany et al., 2020).

The work also has a positive environmental impact. State-of-the-art traffic control systems contribute to reducing vehicle emissions and fuel consumption by making traffic flow more smoothly and reducing stop-and-go conditions (Alvarez Lopez et al., 2019), (Ayeelyan et al., 2022); this is especially important in places like Kuwait, where urban growth as well as economic growth have made traffic worse, which is a big concern for air quality (Helbing et al., 2000). This study demonstrates the real benefits of using intelligent systems in Kuwait by putting the research in the perspective of the country’s unique traffic patterns and infrastructure. These benefits include reducing congestion hot spots and enhancing urban air quality.

The proposed study uses a strict experimental design with multi-seed and multi-demand (Alvarez Lopez et al., 2019) simulations to make sure that the results are strong and can be applied to other situations. This method takes into account the random differences in how vehicles arrive and how drivers behave, which enables us to make statistically sound conclusions about how each control algorithm performs compared to the others. Furthermore, a set of related statistical analyses, including confidence intervals and hypothesis testing, is performed to demonstrate the observed differences, ensuring that the recommendations are based on substantial evidence.

This proposed study enables three significant contributions. First, it fills a large gap in the literature on holistic algorithmic benchmarking (Ault and Sharon, 2021), (Alvarez Lopez et al., 2019), (Almomany and Jarrah, 2024) by giving a detailed comparison of four state-of-the-art traffic control algorithms and an RL-based approach under various demand scenarios. Second, it shows that FPGA-based hardware acceleration for traffic control decision-making is feasible and valuable, offering significant improvements in computational latency that are important for real-time applications. Third, by situating the study within the context of Kuwait’s specific areas, it introduces a functional, constructed approach to utilizing new traffic control systems to address challenges in this area, which could also be applicable in other cities around the world. This study also contributes to the body of knowledge on how intelligent traffic control systems can enhance the quality of life for residents in smart cities by incorporating algorithmic innovation, rigorous simulation, hardware optimization, and local contextual analysis (Michailidis et al., 2025), (Ault and Sharon, 2021), (Alvarez Lopez et al., 2019). It shows how important it is to combine different areas of study, like traffic engineering, artificial intelligence, and hardware design, to come up with solutions that work in both theory and practice, especially in complicated urban settings.

This study investigates four main types of traffic signal control algorithms: Fixed-Time (Muralidharan et al., 2015), Max-Pressure (Varaiya, 2013), Delay-Based (Wu et al., 2018), and a Hybrid approach (Kouvelas et al., 2017). These algorithms range from static scheduling to highly responsive methods that depend on the current state of the system. We make use of the Simulation of Urban Mobility (SUMO) platform, which is a popular tool for modeling and analyzing transportation networks and control strategies. This open-source, microscopic traffic simulator systematically implements the aforementioned algorithms in realistic urban settings (Alvarez Lopez et al., 2019). This section thoroughly describes the design and functionality of each algorithm that enhances the traffic control system. Also, we provide a comprehensive overview of the design and operation of each algorithm employed to enhance the traffic control system.

### 1.1 Advanced traffic signal control algorithms

#### 1.1.1 Fixed-time control

One of the oldest methods in traffic management is fixed-time signal control, in which traffic signals work based on predetermined cycle lengths, phase splits, and offsets. Usually, these numbers are based on past traffic volumes and are only updated occasionally. The most promising aspect of this method is that it’s easy to understand, set up, and maintain. However, it struggles to accommodate real-time traffic fluctuations, which often result in inadequate use of green light durations during times of varying demand levels.

Making use of the well-known Webster’s formula, shown below, that is still widely documented in modern studies (Gartner et al., 2001), a fixed-time control strategy can be mathematically defined by enhancing the cycle length $F_{c}$ and the green times $gn_{t}$ for each stage t , usually by minimizing the average delay per vehicle, as in Equation 1.

$$F_{c}=\frac{1.5\cdot L_{\mathrm{time}}+5}{1-Y},\;g_{nt}=\frac{y_{t}}{Y}\left(F_{c}-L_{\mathrm{time}}\right)$$ (1)

Where $L_{\mathrm{time}}$ is the total amount of time lost per cycle and Y is the sum of the critical flow ratios $y_{t}$ for all approaches. This formulation attempts to keep the flow of traffic through the intersection while balancing delays.

Despite its constraints in dynamic circumstances, fixed-time control remains a standard for comparative investigations. This persistence is due to its extensive historical use and its fundamental role in the design of basic traffic signals (Li et al., 2014). For example, in [(Li et al., 2014)], the authors used fixed-time control as a baseline for testing adaptive systems, demonstrating how much more effective and responsive strategies were at achieving results. This work highlights how classical fixed-time plans serve as the basis for evaluating actuated and adaptive algorithms.

#### 1.1.2 Max-pressure control

In 2013, Varaiya (Varaiya, 2013) proposed a novel approach to traffic signal control known as max-pressure control. This decentralized strategy dynamically selects signal phases to maximize the pressure, defined as the difference between incoming and outgoing vehicle queues, weighted by lane capacities. The method naturally encourages the network to focus on load balancing by prioritizing movements with significant imbalances. For every intersection, the phase ϕ* is mathematically chosen to make this pressure as high as possible. Here, $\mu_{ab}$ is the saturation flow rate from lane a to lane b , and $q_{a}$ and $q_{b}$ are the lengths of the queues.

$$\phi^{*}=\arg\underset{\phi }{\max}\underset{\left(a,b\right)\in \phi }{\sum }\mu_{ab}\left(q_{a}-q_{b}\right)$$ (2)

This formulation yields an emergent property: the network tends to self-stabilize, maintaining low overall queue lengths even under heavy traffic. Extensive research has examined the stability and throughput optimality of this approach. For instance, (Wongpiromsarn et al., 2012) demonstrated that max-pressure control maximizes throughput under certain stochastic demand models. More recently, (Kouvelas et al., 2017) provided empirical evidence of its applicability in urban networks, showing that max-pressure policies outperform static timing plans, particularly in environments with highly variable demand.

#### 1.1.3 Delay-based control

The main objective of delay-based controllers is to minimize the total time that cars have to wait at intersections. While max-pressure strategies examine the lengths of queues, delay-based strategies use real-time estimates to predict vehicle delays. These strategies dynamically adjust the duration of green lights to reduce the overall delay.

To reach this goal, the process of making decisions at each interval includes looking at the following, as in Equation 3:

$$\underset{g_{t}}{\min}\underset{t}{\sum }D_{t}\left(g_{t}\right),and:\underset{t}{\sum }g_{t}\leq C-L$$ (3)

where $D_{t}\left(g_{t}\right)$ is the estimated delay for approach t based on the green time given to it $g_{t}$ , and C is the length of the traffic cycle. People often use cumulative arrival and departure curves or models like the Akçelik delay formula to figure out delay functions.

Papageorgiou et al. (2003) found that adding real-time delay measurements to highly congested networks significantly improves their performance. Lin et al. (2015) also used short-term traffic predictions to help reduce delays, which made the average wait time at intersections even shorter.

#### 1.1.4 Hybrid delay approach

The hybrid delay management approach combines components of both delay minimization and pressure balancing. It adjusts policies based on traffic conditions. When there are little to moderate traffic conditions, the system runs in delay minimization mode to reduce travel times. As traffic becomes more congested, it shifts toward max-pressure or queue balancing to avoid traffic congestion.

Thereby, the hybrid controller effectively addresses the following optimization problems as in Equation 4.

$$\left\{\begin{array}{cc} \arg\min_{\phi }\underset{a}{\sum }D_{a}\left(g_{a}\right),\; & \rho <\rho_{th}\\ \arg\max_{\phi }\underset{\left(a,b\right)\in \phi }{\sum }\mu_{ab}\left(q_{a}-q_{b}\right),\; & \text{otherwise} \end{array}\right.$$ (4)

Where ρ is the level of network congestion (such as the average occupancy) compared to a threshold $\rho_{t}$ , these kinds of hybrid methods are particularly effective even when traffic demand fluctuates.

Diakaki et al. (2002), Michailidis et al. (2025) researches show that hybrid controllers can keep delays low in regular traffic and control spillbacks in heavy traffic. Due to their versatility, these controllers are an excellent choice for addressing various types of city traffic challenges.

#### 1.1.5 Reinforcement learning in traffic signal control

Reinforcement Learning (RL) has become an effective tool (Gabler and Wollherr, 2024), (Pham et al., 2025) for improving traffic signal control by enabling an agent to develop adaptable strategies that reduce congestion and delay (Agrahari et al., 2024). RL frameworks do not rely on predefined traffic patterns, unlike traditional methods. Instead, they learn optimal ways to control traffic by interacting with the environment (Wang et al., 2024). The intersection control problem is usually modeled with a Markov Decision Process (MDP). This model encompasses various states, including the length of a queue and the number of people in a room, as well as decisions such as changing the phases of a signal and rewards designed to reduce wait times or increase throughput. Recent studies have indicated that RL works very well in traffic situations that are constantly changing and challenging to predict. For instance, in (Saadi et al., 2025), the authors demonstrated how the RL approach can be applied in traffic control management, significantly reducing delays compared to fixed-time or actuated systems. They demonstrate that it can reduce delays by a significant factor compared to fixed-time or actuated systems. In an existing study (Van der Pol and Oliehoek, 2016), the authors also demonstrated that deep Q-learning methods can change traffic lights to fit patterns of congestion that do not happen frequently; this makes the whole system work much better. These improvements make RL a promising approach to developing innovative traffic management systems that can adapt in real-time, thereby helping to ease congestion in cities.

A Markov Decision Process (MDP) is often used to describe the problem of traffic signal control. It is defined by the tuple (S,A,P,R,γ) , where:

-
S represents an entire set of states, including the number of cars on incoming lanes and the total length of the queues.
-
A is the set of prospective actions, such as determining the next traffic phase and how long that the green light remains on.
-
$P\left(s^{\prime }|s,a\right)$ represents the state transition probability, which indicates how likely it is that you will move from state s to state $s^{\prime }$ when action a is taken.
-
R(s,a) represents the immediate reward obtained after taking action a in state s ; this reward is normally designed to minimize total delay or queue length.
-
γ∈[0,1] is the discount factor, indicating the significance of future rewards.

The goal is to identify a policy π:S→A that optimizes the anticipated cumulative discounted reward over time as described in Equation 5.

$$\underset{\pi }{\max}\;E\left[\overset{\infty }{\underset{t=0}{\sum }}\gamma^{t}R\left(s_{t},a_{t}\right)\right]$$ (5)

where the action $a_{t}=\pi \left(s_{t}\right)$ is chosen according to the policy at time step t .

The rest of this proposed research study is organized in the following order: The investigation of the recent research on advanced traffic management is handled in Section 2, and FPGA high-speed computation platform is discussed in Section 3. In Section 4, the methodological framework is laid out, including the simulation models, traffic demand scenarios, and hardware design processes. Section 4 illustrates and discusses the experimental results, including the performance of the algorithms with varying traffic loads and the significant speedup achieved with FPGA acceleration. Section 5 examines the practical implications of this for real-world use in Kuwait, focusing on fuel savings and reduced emissions. Finally, Section 6 wraps up the paper by listing the main findings, the study’s limitations, and recommendations for future research.

## 2 Literature review

Over the past 5 years, numerous researchers have investigated the implementation of advanced traffic signal control systems to mitigate traffic congestion in cities. Recent studies have increasingly utilized data-driven methods, such as reinforcement learning (RL) and deep learning, to develop adaptive traffic light strategies that outperform fixed-time or actuated controls. In a related study (Michailidis et al., 2025), the authors proposed a thorough review that demonstrates how RL frameworks enable traffic controllers to learn the most effective policies from immediate changes in traffic state, thereby making them more responsive to unpredictable demand. In a parallel manner, the authors in (Ayeelyan et al., 2022) demonstrated that incorporating experience replay and target networks into deep RL architectures yields significant reductions in average delays at junctions.

Researchers have also investigated strategies that combine classical queue-based or pressure-based models with learning methods; these are similar to algorithmic improvements. The authors of Kouvelas et al. (2017) indicated that networks can stay stable no matter how much traffic there is by using max-pressure logic and local delay minimization together. In Lin et al. (2015), the authors employed predictive control strategies that utilize short-term traffic forecasts to make the flow smoother and reduce spillbacks simultaneously.

Alvarez Lopez et al. (2019) enhanced the SUMO simulation framework to increase its scalability, allowing it to accommodate a range of demanding experiments. This enhancement made it possible to test traffic management algorithms using statistics rigorously; these features have been essential to evaluate RL-based and hybrid controllers in real-world situations with stochastic vehicle arrivals.

At the same time, the application of hardware solutions is another crucial area of research. According to Banerjee (2024), real-time applications can be made achievable even in high-traffic environments by drastically lowering latency through the development of intelligent traffic light controllers on FPGA devices. This line of investigation underscores the importance of computational efficiency in delivering workable and scalable solutions. The design of controllers that can function effectively in various urban networks while maintaining performance in the presence of sensor noise and erratic driver behavior remains a challenge. Innovation in city traffic management solutions is driven by the necessity to address these issues. Table 1 gives a short summary of important studies from the last 10 years that used AI to control traffic lights. It describes the methods used in each study, such as reinforcement learning, deep learning, or multi-agent systems, as well as the main results and any improvements in performance that were reported. This summary puts the proposed method in the context of other research and shows how AI is becoming more and more important for improving urban mobility.

**TABLE 1:** Recent AI-Based traffic signal control approaches and their performance outcomes.

Study/Year	Approach	Key contribution	Results	References
Tan et al. (2019)	Deep RL (DQN)	Applies DRL with realistic traffic scenarios	+47% delay reduction vs. fixed-timing	Tan et al. (2024)
Gao et al. (2017)	Deep RL w/experience replay	Learns from raw traffic data	Up to 86% reduction vs. fixed	Gao et al. (2017)
Agrahari et al. (2024)	Review paper	AI techniques for adaptive TSC	Taxonomy of RL, DRL, fuzzyetc.	Agrahari et al. (2024)
Xiao (2025)	Reinforcement Learning	Highlights RL benefits in TSC	Improved flow	Xiao (2025)
Othman et al. (2025)	Multi-agent RL	Decentralized multimodal control	Person-delay optimized	Othman et al. (2025)

## 3 FPGA technology

Field Programmable Gate Arrays (FPGAs) have become powerful tools for spatially reconfigurable computing. They have been used successfully in many areas, including pattern recognition, image processing, signal processing, real-time control systems, networking, machine learning, cybersecurity, and cyber-physical systems (Almomany et al., 2022a). This technology enables the possibility of dynamically changing control logic and data paths at a very fine level, even while the program is running. This means that hardware configurations can be very closely matched to the time and algorithmic needs of particular applications (Almomany and Jarrah, 2024). Because of this, FPGA-based solutions can attain close to the high performance and low energy use of dedicated ASICs while still being as flexible as software implementations on general-purpose multi-core CPUs (Almomany et al., 2020). Three main types of FPGA-based spatially reconfigurable environments are popular in business: commodity FPGA accelerator cards, stand-alone System-on-Programmable-Chip (SOPC) systems, and new cloud-based FPGA platforms. Accelerator cards, which are often used as PCIe add-ons, are designed for high performance and include high-end FPGAs with extensive local DDR memory. They also often come with high-speed networking and flash storage for configuration. SOPC systems, on the other hand, have both embedded processors and FPGAs, making them stand-alone computing platforms. Cloud providers now offer FPGA resources that can be managed through virtualized infrastructures, such as OpenStack, making them more widely available (Almomany et al., 2022b). FPGA deployments have some benefits, but they also have significant drawbacks. For example, it can be hard to optimize time-shared hardware resources, and there are long reconfiguration delays—sometimes lasting seconds—because their internal clock speeds are over 300 MHz (Almomany and Jarrah, 2024), (Almomany et al., 2022a).

## 4 Methodology and simulation environment

For this study, we used the Simulation of Urban Mobility (SUMO) (Erdmann, 2015) to investigate the four distinct approaches of regulating traffic signals: Fixed-Time, Max-Pressure, Delay-Based, and a Hybrid Delay/Max-Pressure approach. Utilizing the proposed grid tool, the netgenerate, we created a regular grid network with 16 intersections, each with two lanes in each direction and 300 m of road between them. We used the “–tls.guess” option to automatically add traffic lights at each intersection, making the cross-junctions appear more realistic, like those found in real cities.

The SUMO tool, known as randomTrips.py, generates random traffic demands by creating trips for vehicles between random pairs of origins and destinations throughout the entire network. We established three levels of traffic demand to make sure that there were a variety of congestion situations:

- Low demand: 1200 trips per hour (every 3 s).

• Medium demand: trips every 2 s (about 1800 trips per hour).
- High demand: trips every 1 s (about 3600 trips per hour).

We ran each level of demand with several different independent seeds (42, 123, 2025, 5555, and 9999) to account for the random changes in the number of vehicles arriving and the amount of network congestion.

We used the TraCI API to connect each control strategy to SUMO through a Python script. The script altered the phases of the traffic lights according to the algorithm. The Fixed-Time controller made sure that each phase had a fixed cycle of 30 s. The Max-Pressure controller selected the phase with the most significant difference between the lengths of the incoming and outgoing queues. It did this at each timestep. The Delay-Based controller prioritized phases with the longest lane delays, while the Hybrid controller utilized both pressure and delay heuristics with configurable thresholds.

The Max-Pressure algorithm was parallelized and written in VHDL to work with FPGA platforms, aiming to explore hardware acceleration. This design utilizes parallel comparators and counters to achieve intersection decision latencies of under 2 ns, which is significantly faster than CPU micro-benchmarks that average 37 ns per intersection decision.

All of the simulations kept track of critical data, such as the number of vehicles waiting, the average lane occupancy, and the amount of $CO_{2}$ emissions. They did this over 3600 simulation steps, which is the same as 1 hour of traffic flow. The data was then stored in structured CSV files for later analysis. Table 2 gives a full list of the simulation parameters and experimental setups used in this study. We adjusted the frequency of trips to evaluate the effectiveness of controllers under three different road conditions: low, medium, and high. To assess the robustness of the results, multi-seed experiments were conducted by systematically varying the random seed in traffic generation. This approach enabled the possibility of accomplishing statistical analysis on different types of traffic realizations. We developed each control approach as a Python script that operates in real-time with SUMO, adjusting the phases of traffic signals according to the logic of each approach. At the same time, a Max-Pressure controller architecture was designed in VHDL to run on FPGA platforms, aiming to explore hardware acceleration. This demonstrated that the latency of calculations was significantly reduced and the throughput was increased compared to CPU-based implementations. Additionally, SUMO’s emission modules were utilized to track $CO_{2}$ emissions, allowing for the simultaneous investigation of both environmental and traffic effects. Stop-and-go traffic at signalized intersections, frequent speeding up and slowing down, and long periods of idling are the main causes of $CO_{2}$ emissions. Our proposed adaptive signal control method cuts down on fuel consumption and, as a result, $CO_{2}$ emissions by cutting down on the time cars spend idling and improving the timing of green light phases.

**TABLE 2:** Summary of experimental configurations.

Parameter	Value
Grid size	4×4 intersections (16 total)
Road length	300 m per segment
Lanes	2 lanes per direction
Traffic lights	Automatically generated with –tls.guess
Demand levels	Low (3 s/trip), Medium (2 s/trip), High (1 s/trip)
Seeds used	42, 123, 2025, 5555, 9999
Simulation duration	3600 steps (1 h)
Metrics logged	Total waiting, mean waiting, ${\text{CO}}_{2}$ emissions

**FIGURE 1:**
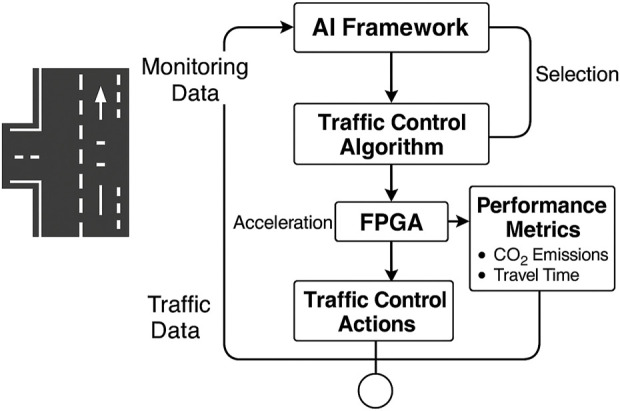
Intelligent AI-FPGA-Integrated Framework for Adaptive Traffic Signal Control. The system monitors road conditions, utilizes AI to select an appropriate algorithm according to aspects such as emissions and wait times, and accelerates control execution on FPGA hardware.

Figure 1 illustrates an innovative framework for the suggested innovative traffic management system. The system utilizes an AI-based selector to continuously monitor real-time road conditions, including the number of vehicles and the length of queues. Then it selects the most effective method for controlling traffic. This selector examines several factors, including total wait time, $CO_{2}$ emissions, and queue statistics, to identify the algorithm that works best. Then, the chosen traffic control logic, which can be Max-Pressure, Delay-Based, Hybrid, or any other advanced reinforcement learning method, is implemented on FPGA hardware so that it can respond in real-time. The FPGA then sends control signals to traffic lights, enabling the system to adapt and enhance traffic flow in response to changing demand levels, all facilitated by continuous feedback loops. The FPGA-based computing design can construct an efficient pipelined architecture that enables the overlapping of multiple instructions, allowing more operations to be executed within each clock cycle. This architectural choice is particularly beneficial for applications with a real-time requirement. Furthermore, the design’s scalability benefits from the flexible resources offered by FPGAs; higher-capacity devices can support more complex implementations (Almomany et al., 2020). By utilizing FPGAs with greater resources, the system can be extended to handle more sophisticated designs. This adaptability ensures that the system can meet growing computational demands while maintaining real-time responsiveness (Almomany et al., 2024). Numerous studies have evaluated the cost-effectiveness of using field programmable gate arrays (FPGAs) as computing platforms to reduce energy consumption while meeting real-time performance requirements. For instance, (Qasaimeh et al., 2019) highlight the significant benefits of FPGAs in real-time embedded applications, especially in signal and image processing. Compared to traditional CPUs and GPUs, FPGAs offer greater parallelism, lower latency, and hardware-level reconfigurability, enabling more efficient execution of complex computations. These features make FPGAs particularly suitable for embedded systems operating under strict resource constraints. Additionally, FPGAs exhibit deterministic behavior and superior energy efficiency—critical advantages for time-sensitive applications such as real-time traffic control in smart cities. Further research supports their applicability in edge computing, where consistent throughput across varying workloads, architectural flexibility, and fine-grained parallelism are essential. Notably, FPGAs demonstrate 3—4 times lower power consumption and up to 30.7 times higher energy efficiency compared to GPUs (Biookaghazadeh et al., 2018), making them ideal for energy-constrained IoT environments. FPGAs come in different classes with varying resource capabilities. Standalone FPGA boards, which are well-suited for commercial and academic use, typically cost between $200 and $800. More advanced boards designed for complex applications may range in the thousands of dollars [Ref]. While the initial cost of FPGA platforms may exceed that of general-purpose microcontrollers or GPUs, their long-term advantages—such as energy savings, reusability, and reliability—can outweigh the upfront investment. As noted by Maschi et al. (2021), integrating FPGAs into large-scale commercial systems may require software adaptations and infrastructure realignment, potentially increasing initial costs. However, in smart city and IoT deployments, their low power consumption, reconfigurability, and long operational lifespan make FPGAs a cost-effective and sustainable solution, particularly for cities with limited budgets (Ramamoorthy, 2025).

## 5 Results and discussion

**FIGURE 2:**
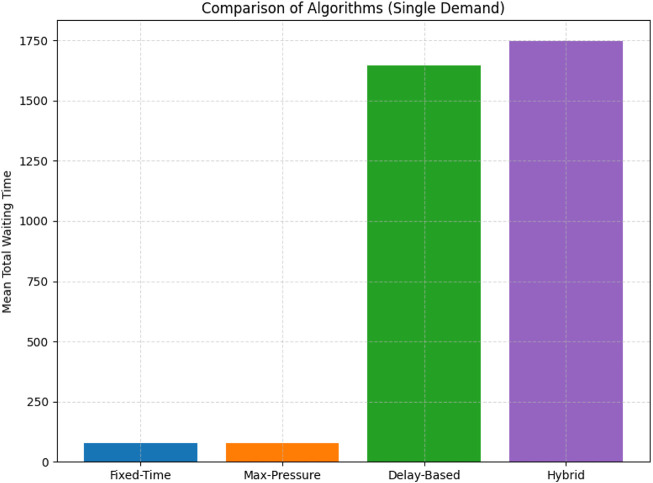
Initial single-demand results showing significantly higher waiting times for Delay and Hybrid controllers versus Fixed-Time and Max-Pressure under 3600 vehicle scenario.

**FIGURE 3:**
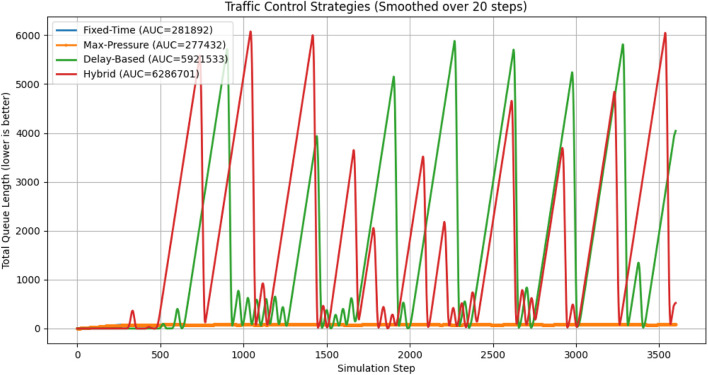
Initial comparison of total waiting time across Fixed-Time, Max-Pressure, Delay-Based, and Hybrid controllers on baseline scenario.

We used the SUMO traffic simulator to develop and evaluate four traffic control algorithms in the first part of this study: Fixed-Time, Max-Pressure, Delay-Based, and a Hybrid strategy. Our initial attempts were conducted with a steady traffic demand of approximately 3,600 vehicles, following a static pattern. Under these circumstances, the results clearly showed that Delay-Based and Hybrid controllers performed significantly worse than Fixed-Time and Max-Pressure strategies in terms of both total waiting time (Figure 2) and the accumulated area under the curve (AUC) of waiting vehicles (Figure 3). The Delay-Based and Hybrid strategies had mean waiting times that were too high, with more than 1600 vehicles waiting. In contrast, the Fixed-Time and Max-Pressure strategies maintained their values at significantly lower levels, around 78 and 77, respectively.

The significant variation observed can largely be attributed to the fact that the early Delay and Hybrid controllers had naive parameter settings, such as overly conservative thresholds or basic delay estimates, which made the phase switching less responsive. As a result, Fixed-Time and Max-Pressure maintained stable cycle executions, even when traffic loads were consistently low, effectively preventing queues from growing too quickly. These insights informed the addition of adaptive logic to the final design, allowing the system to monitor real-time queue lengths and phase delays. This enables it to switch to more stable algorithms, such as Max-Pressure or Fixed-Time, when the Delay or Hybrid controllers are not functioning as expected.

We realized that static, single-demand simulations do not accurately reflect how fundamental urban traffic changes. To address this, we introduced three demand levels–Low, Medium, and High–and ran multiple seeds to introduce random variability. This approach created a variety of trip distributions to more closely mimic real-life traffic situations. The multi-seed trials showed that the variations in performance between each of the algorithms decreased significantly when the conditions were more realistic. For instance, under high demand, the average wait times for all controllers stayed close to 80 vehicles, with standard deviations less than 1.0.

**FIGURE 4:**
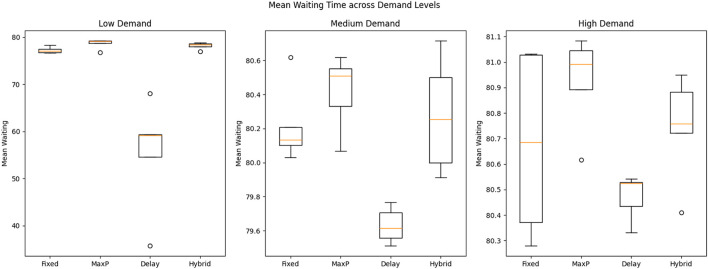
Mean waiting times under low, medium, and high traffic demand levels, comparing all four algorithms.

Table 3 and Figure 4 present these combined results, which confirm that adjusting delay thresholds and hybrid switching strategies significantly improved performance during fluctuating traffic conditions. These enhancements demonstrate the framework’s robustness in maintaining smooth traffic flow under unpredictable demand patterns.

**TABLE 3:** Mean waiting times (vehicles) under multi-seed experiments.

Algorithm	Low demand	Medium demand	High demand
Fixed-Time	77.27±0.60	80.22±0.21	80.68±0.32
Max-Pressure	78.62±0.96	80.42±0.20	80.93±0.17
Delay-Based	55.37±10.75	79.63±0.09	80.47±0.08
Hybrid	78.20±0.68	80.28±0.30	80.74±0.19

**FIGURE 5:**
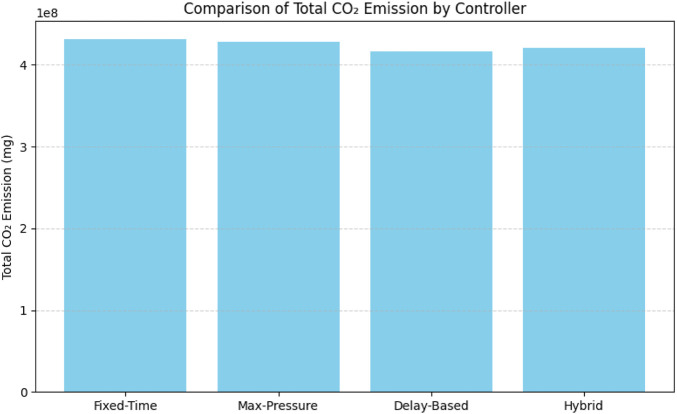
Comparison of total ${\text{CO}}_{2}$ emissions under different traffic control algorithms.

This study established an emission framework to estimate the quantity of $CO_{2}$ emissions from automobiles under each proposed traffic control strategy in order to evaluate the strategies’ environmental effects. The total emissions were figured by incorporating immediate fuel consumption and speed-dependent emission rates for all cars, using approaches comparable to those found in the Motor Vehicle Emission Simulator (MOVES) developed by the U.S. Environmental Protection Agency,and Handbook Emission Factors for Road Transport (HBEFA) frameworks (Erdmann, 2015), (Barth and Boriboonsomsin, 2000) that are commonly used in Europe. These models link the speed and acceleration information of vehicles to emission indicators, which lets us roughly measure emissions in the proposed traffic simulations. In our tests, we found that the total CO2 emissions from the different control algorithms were broadly similar; this means that none of the approaches delivered significant variations in overall emission rates given the demand scenarios and cycle lengths we employed. Figure 5 shows how much $CO_{2}$ each algorithm produces in comparison to the others.

It is important to note, however, that although our specific results did not show an apparent decrease, numerous studies have demonstrated that reducing traffic congestion generally leads to improved air quality and lower greenhouse gas emissions by reducing idle times and making stop-and-go driving patterns smoother (Cui, 2025), (Jiang et al., 2017); Thus, innovative traffic management systems are crucial not only for enhancing mobility but also for significantly benefiting the environment. Recent advancements reveal that the environmental benefits and capacity enhancements of intelligent traffic control systems can be significantly improved by the incorporation of Connected Automated Vehicles (CAVs). For example, (Qin et al., 2024) created an analytical model for mixed traffic at unsignalized priority intersections that included both connected automated vehicles (CAVs) and regular vehicles (RVs). Their results show that more CAVs, better headways, and platoon formation all make minor roads much more useful. These insights show that FPGA-accelerated adaptive control systems and new CAV technologies could work together to make urban intersections more efficient while also reducing emissions.

**FIGURE 6:**
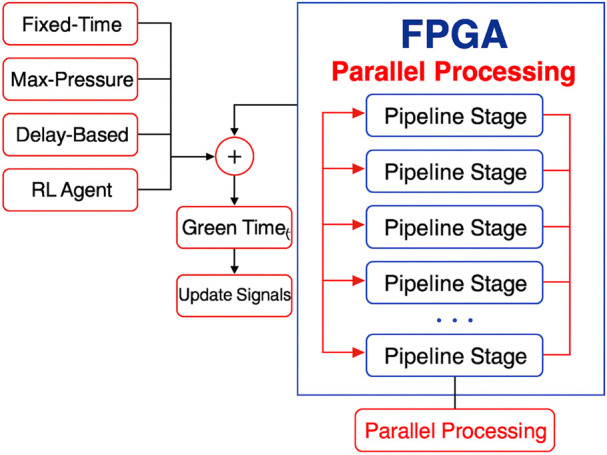
FPGA-based computation platform with custom-designed pipeline stages. This architecture enables overlapping of multiple computations, allowing more operations to be completed per clock cycle. As a result, the total number of clock cycles required to execute one full task is significantly reduced, thereby enhancing the system’s overall efficiency and throughput.

This study presents an FPGA-accelerated architecture that executes traffic control computations in parallel, significantly reducing decision latency. This comes in addition to improvements to the algorithms. The constructed hardware solution utilizes parallelism and pipeline construction to expedite decision-making for all four traffic control algorithms, as shown in Figure 6.

**FIGURE 7:**
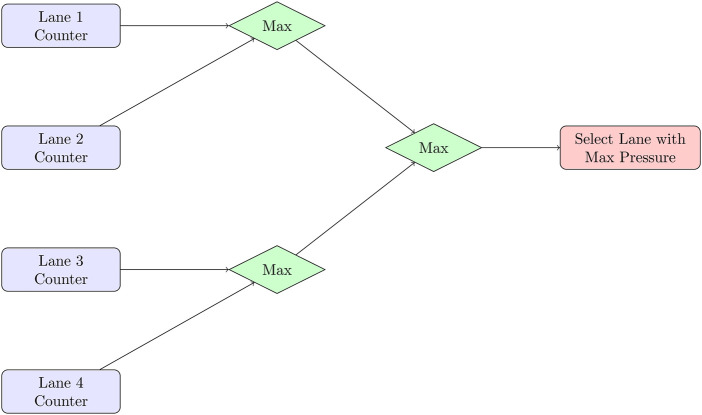
FPGA pipeline for Max-Pressure control: parallel lane counters feed a comparator tree, selecting the highest pressure lane in approximately three stages.

For example, the Max-Pressure algorithm was run on an FPGA with parallel lane counters that fed a multi-stage comparator tree. This resulted in a fully pipelined architecture with approximately three stages for an 8-lane intersection as shown in Figure 7. This allows one decision to be made at each intersection every clock cycle after the pipeline is complete, resulting in a total latency of approximately 15 ns on a 200 MHz device.

As shown in Table 4, Fixed-Time had been translated into a periodic state machine that required only one stage (latency ≈ 5 ns). Delay-Based and Hybrid controllers used shared counters and more threshold comparators with 2–4 pipeline stages. The FPGA is approximately 2–7 times faster than the Apple M4 Max CPU baseline, which results in an average of 36.78 ns per decision. It also guarantees deterministic throughput.

**TABLE 4:** Estimated FPGA pipeline latencies and speedups over the CPU implementation.

Algorithm	Pipeline stages	Latency (ns)	Speedup vs. CPU
Max-Pressure	3	≈15	2.5×
Fixed-Time	1	≈5	7×
Delay-Based	2	≈10	3.5×
Hybrid	4	≈20	1.8×

**FIGURE 8:**
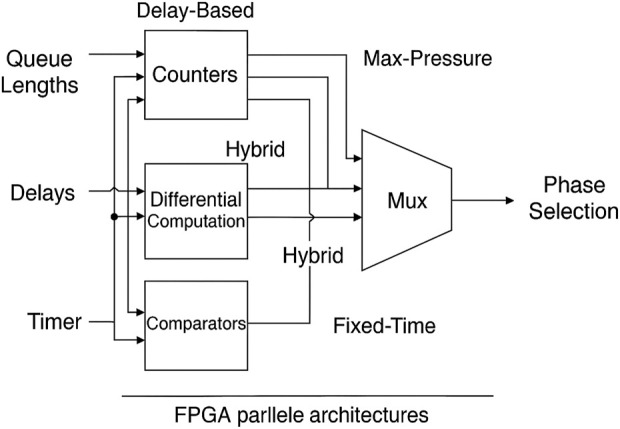
FPGA parallel Architecture.

These results demonstrate that FPGA acceleration is beneficial not only for the Max-Pressure controller, which requires substantial processing power, but also for simpler algorithms, where the FPGA ensures the system operates at high speed consistently, regardless of the demand. In the Max-Pressure approach, the constructed design has a multi-level comparator tree to determine the highest level of differential pressure. On the other side, the Delay-Based controller used parallel delay accumulators for each lane, followed by threshold comparators. The Fixed-Time controller was linked to a minimal period counter, which only needed one pipeline stage. For the Hybrid controller, a single architecture was created that used shared parallel counters for both queue lengths and delays. Based on real-time traffic levels, multiplexers determined the best strategy, allowing them to switch between delay-based thresholds and pressure-based decisions. Figure 8 illustrates the proposed approach for parallelizing and implementing the constructed design on an FPGA.

**FIGURE 9:**
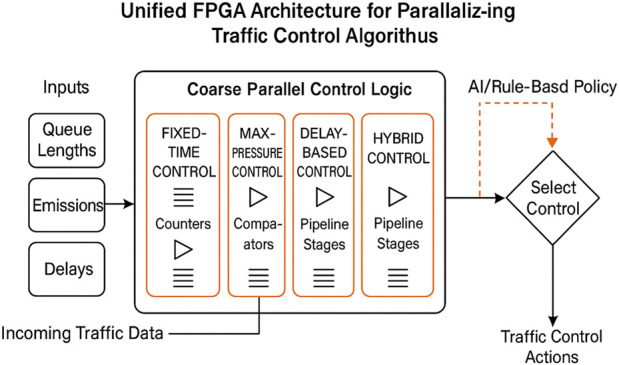
Unified FPGA architecture for parallelized traffic control algorithms.

In Figure 9, the design reveals a parallel pipeline structure for Fixed-Time, Max-Pressure, Delay-Based, and Hybrid controllers. These controllers all process incoming traffic data streams simultaneously. A selection mechanism, which takes into account current traffic conditions, determines which control strategy to employ. This approach significantly reduces decision latency and facilitates multi-metric optimization, including delay, ${\text{CO}}_{2}$ , and throughput.

We first modeled the FPGA VHDL modules in Python to ensure they were correct. Then, we utilized high-level synthesis (HLS) frameworks to convert them into synthesizable VHDL automatically. This made it easy to look into pipeline depths and trade-offs in resources quickly. We used ModelSim to simulate the IP cores and sent them to a Kintex-7 device to examine the execution time.

While using the FPGA high-speed computation platform offers several advantages, its practical deployment presents multiple challenges. The high initial cost of these dedicated platforms, along with the need for more expertise in hardware description languages and underlying hardware, can be a major concern; this expertise is required as the process of optimization to create a more efficient design requires such knowledge. The integration with data acquisition devices and adapting to new smart sensor requirements may also demand additional effort to address compatibility and reliability issues. To overcome these challenges, city planners and technology providers must collaborate to ensure that new TMS are implemented in a way that is both environmentally friendly and cost-efficient.

### 5.1 Kuwait relevance

Kuwait has a hard time getting around cities because it is so small and there are so many cars on the road. According to NationMaster, Kuwait has approximately 527 cars for every 1,000 people, which is significantly more than the global average of around 182 cars per 1,000 people (List of countries and territories, 2024). It is also higher than the number of vehicles in some non-Gulf countries, such as India (India (158 vehicles per 1,000) vehicles for every 1,000 people) (List of countries and territories, 2024). The high rate of motorization, combined with a rapidly growing population (most of whom reside in cities), has made traffic congestion a persistent issue on major roads, including the Fifth Ring Road, King Fahd Highway, and Airport Road, particularly during rush hour.

This traffic congestion has repercussions that extend beyond simply hindering drivers. Kuwait’s air quality is still a big problem for both the environment and people’s health. The World Health Organization says that the safe level of ${\text{PM}}_{2.5}$ in Kuwait City is $5\;\mu \;{g/m}^{3}$ . However, the levels are always higher than that, averaging between $30--46\;\mu {g/m}^{3}$ (IQAir, 2023). Natural sources like dust storms add to particulate matter, but studies show that vehicular emissions are the leading human-made cause of air pollution in cities across Kuwait (Elmi and Al Rifai, 2012). When traffic is heavy, cars have to stop and go more frequently, which increases ${\text{CO}}_{2}$ and ${\text{NO}}_{x}$ emissions and worsens air quality. It also uses more fuel.

In this situation, using a intelligent, adaptive traffic control system that selects from several algorithms based on real-time demand conditions, like the one suggested in this study, looks like a good way to solve the problem. The system can make decisions in under a second by utilizing advanced traffic signal control logic on high-performance FPGA hardware. This enables the implementation of faster and more precise adjustments to traffic signal timings, resulting in smoother traffic flow, shorter lines, and reduced wait times for cars at intersections.

The proposed solution has a multi-algorithm architecture that includes Fixed-Time, Max-Pressure, Delay-Based, and Hybrid approaches. This implies it can tolerate a wide range of traffic styles well. It can change to fit different situations, which makes it perfect for addressing the problems of a city like Kuwait City. Additionally, the system’s ability to adapt to changing traffic needs makes it a scalable foundation for long-term, creative urban planning projects. It helps the environment and public health by lowering emissions and fuel use, and it also makes it easier for people to get around.

## 6 Conclusion & future work

This study demonstrates a unified and adaptable traffic control framework that aims to make smart cities more mobile, less congested, and better for environmental health. The study demonstrates that dynamically selecting the best control method—Fixed-Time, Max-Pressure, Delay-Based, or Hybrid—based on real-time traffic conditions improves performance. We examined the proposed system using practical modelling tools and rigorous statistical tests to ensure that the proposed design could handle varying levels of demand. Furthermore, utilizing a high-speed FPGA as a hardware computation platform can significantly accelerate the entire process, ensuring that it meets the real-time requirements. The proposed solution held considerable promise for minimizing the traffic congestion on major roads in Kuwait, reducing fuel consumption, and enhancing air quality. The proposed framework not only facilitates easier mobility for individuals but also lays the groundwork for scalable, energy-efficient traffic management systems that align with the objectives of new urban development. The ability to adaptively optimise signal control in response to altering traffic loads overcomes a long-standing problem with conventional fixed-time strategies; this demonstrates the benefits of combining algorithmic flexibility with a hardware-level solution. In comparison to modern high-performance CPUs, this study suggests an effective, real-time traffic control system design that combines reinforcement learning algorithms with FPGA-based acceleration, resulting in a speedup of more than seven times. The proposed framework dynamically chooses the best traffic control strategies, which significantly reduce congestion and $CO_{2}$ emissions while ensuring that hardware runs efficiently. While promising, there are some problems that need to be worked out, such as the need for real-world deployment and testing for scalability across larger networks. Overall, this work offers a flexible and low-latency solution for smart cities, setting the stage for more research on coordinated multi-agent control and smooth integration with urban IoT systems.

Subsequent research will expand upon this study to synchronize traffic throughout the city, entailing the creation of a network of interconnected traffic signals capable of real-time data sharing. This expansion will enable global optimization, rather than making decisions based on local conditions. We will utilize advanced predictive modeling that leverages historical traffic data and real-time sensor inputs to forecast how traffic will flow on the city’s roads. The planned system will utilize machine learning to suggest alternative routes in real-time, thereby reducing traffic throughout the city while balancing travel time, environmental impacts, and network capacity. Moving from isolated adaptive control to holistic, predictive traffic management is a crucial step toward creating smart cities that are sustainable without the threat of traffic jams. We will conduct additional experiments to assess the effectiveness of the proposed system in conjunction with wearable technologies and smartwatches. These efforts will enable drivers to receive real-time alerts and personalized route suggestions directly on their devices. The goal of this integration is to enhance situational awareness, expedite reaction times, and facilitate the implementation of adaptive rerouting strategies.

## Data Availability

The original contributions presented in the study are included in the article/supplementary material, further inquiries can be directed to the corresponding author.
